# Medical Items

**Published:** 1917-09

**Authors:** 


### MEDICAL ITEMS

### FOR THE CLOGGED LIVER.

When the liver does not act as it should, the zest of life departs, and the saying that ''life and living depend upon the liver,'' although somewhat facetious, contains more than a modicum of truth. An engorged liver, of course, signifies that the organ requires certain stimulation, especially when the condition is attended by manifestations of auto-toxemia. If any one fact has been more definitely established than another it it that such stimulation should not be brought about by the use of drastic cathartics, for if so, the remedy is frequently worse than the disease in its sinister effects. What is particularly needed is a means of stimulation which will satisfactorily increase the functional activity of the liver, without setting up catharsis or over activity of the bowels.

The above needs are well met by Chionia, an exceedingly effective and reliable preparation of Chionanthus Virginica. This well know product exerts a distinctly specific action on the liver and is probably one of the most efficient remedies at the command of the physician for stimulating the hepatic function. Administered in regular and appropriate dosage it increases the flow of bile, relieves congestion of the biliary passages, promotes digestion and although it cleanses the intestinal canal is accomplishes this without purging or griping.

Chionia, therefore, has proven of extraordinary value in the treatment of all functional disorders of the liver, especially those grouped under the vague term ''biliousness' and characterized by digestive disturbances, jaundice, constipation, diarrhea, headaches, and auto-toxic symptoms generally. The prompt and decided results that uniformly follow the use of Chionia furnish convincing evidence of the utility of this trustworthy product.

---

### TREATMENT OF HAY FEVER.

Notwithstanding the many "specifics" and "near-specifics" for hay fever that have been pushed forward in recent years, the disease, if not precisely enigmatical, continues to baffle and perplex. It is evident that no single therapeutic agent has arisen that can eliminate, or even modify, the symptoms in all cases. Individual sufferers present problems that are peculiar to themselves, and other than the vasomotor relaxation of the upper respiratory tract, which is common to all, there are no uniform underlying pathologic changes.

Fortunately there are some very satisfactory alleviants. The suprarenal substances, in the form of its isolated active principle, Adrenalin, is undoubtedly one of the best of these. Experienced practitioners say that in a large majority of cases it successfully controls the sypmtoms. Chloride Solution and Adrenalin In halents are the preparations commonly used, being sprayed into the nares and parynx. The former should firest be diluted with four to five times its volume of physiologic salt solution. The latter may be administered full strength or diluted with three to four times its volume of olive oil.

Another agent of large promise in the treatment of autumnal hay fever is Ragweed Pollen Extract. Its strength is based upon the generally accepted theory that this type of hay fever, with occasional exceptions, is due to the pollen of ragweed. An accurately standardized product is supplied by Parke, Davis & Co. It is administered hypodermically.

### HEART TROUBLES.

Numerous persons, especially those of middle age and past and who live a sedentary life, suffer from worrying heart symptoms. As a rule, no organic lesions can be detected but the functional disturbances which are generally in evidence, are a source of constant alarm. Oftentimes, a person's life is made a burden by the pain and other sensations which affect the heart.

Such cases give the physician an infinity of bother. In the first instance, the patient's manner of living must be regulated, appropriate diet must be prescribed and excessive indulgence in narcotics or stimulants must be interdicted.

A regimen of this nature, however, while essential will not effect a complete cure. A therapeutic remedy which will give tone to the tired heart, but which will not act as a spur is needed. The heart requires persuasion instead of driving. Cactina Pillets will not only effect this object but possess the great advantage over the majority of heart remedies—that they have no cumulative action. Consequently, there is no safer heart tonic known than Cactina Pillets. In all cases of functional heart affections their use is strongly indicated, for they unfailingly bring back the heart's action to its normal rhythmical ebb and flow and the patient's fears vanish accordingly.

